# The Pattern of Cardiac Diseases at the Cardiac Clinic of Jimma University Specialised Hospital, South West Ethiopia

**DOI:** 10.4314/ejhs.v20i2.69435

**Published:** 2010-07

**Authors:** Belete Habte, Fessahaye Alemseged, Dawit Tesfaye

**Affiliations:** 1 Department of Internal Medicine, Jimma University, P.O. Box : 1274, E-mail: beletehabte@gmail.com; 2 Department of Biostatistics and Epidemiology, Jimma University

**Keywords:** Cardiac diseases, Pattern, Jimma

## Abstract

**Background:**

Rheumatic heart disease is the commonest cardiac disease in most sub-Saharan African countries, followed by hypertensive heart disease which is rising along with the other non-communicable diseases. However the pattern in our setting is not known. This study aimed to determine the pattern of cardiac diseases among adult patients on follow-up at the cardiac follow-up clinic of Jimma University Specialized Hospital.

**Methods:**

A cross-sectional study was conducted on cardiac patients who are newly enrolled to the cardiac follow up clinic of Jimma university specialized hospital during a five year period from 2003 to 2008. Out of the total 837 cases that were newly enrolled to the clinic in the five year period, 781 patients who had complete record about etiologic diagnosis were included in the study. The data were collected using structured record review checklist. The collected data were then analyzed using SPSS for windows version 12.0.

**Results:**

Rheumatic heart disease was the diagnosis in 256 (32.8%) of the cardiac cases on follow-up followed by hypertensive heart disease and cardiomyopathy accounting for 189 (24.2%) and 158 (20.2%) of cases, respectively. Among Rheumatic heart disease patients; male to female ratio was 0.86:1 and the mean age was 31.4 years. One hundred ninety three (75.4%) of the cases with rheumatic heart disease had echocardiographic report that showed valve(s) involvements of pure MS in 99 (51.3%) and combined MS, MR in 49 (25.4%). Overall, hypertension contributed for a total of 241 (30.9%) of cardiac patients that included 189 (24.2%) hypertensive heart disease and 52 (6.7%) as one major risk factor for ischemic heart disease.

**Conclusion:**

Rheumatic, hypertensive and cardiomyopathic heart diseases accounted for more than three-quarters of cardiac diseases in the study population. This study highlighted the need for further study to determine the burden at community setting.

## Introduction

Epidemiologic transition which is taking place in every part of the world, among all races, ethnic groups, and cultures has resulted in the global rise in cardiovascular disorders (CVD). Today CVD became the most common cause of death accounting for 30% of deaths worldwide, with 80% of the burden now occurring in developing countries. Although human immunodeficiency virus/acquired immunodeficiency syndrome (HIV/AIDS) is the leading overall cause of death in the sub Saharan region, CVD is the second leading killer and is the leading among those over the age of 30 year (1,2.

Cardiac disease as one of the CVD accounted for significant cases of patient visits although the burden for specific etiologic type varies remarkably between regions of the world, which is largely due to the epidemiologic transition and the different stages in its evolution that a country has to undergo. During the course of cardiac disease a subset of them develop heart failure. The vast majority of heart failure causes in sub-Saharan Africa are due to the major non-ischemic causes; with rheumatic heart disease (RHD), hypertensive heart disease (HHD), and cardiomyopathy accounting for over 75% of cases in most series (1, 3, 4, 5).

RHD is estimated to exist with a prevalence of at least 15.6 million cases, with 282,000 new cases and 233,000 deaths each year (6).The incidence of rheumatic fever had declined in the developed countries. However, RHD are still common and evidence suggested that there is little if any decline in the occurrence of rheumatic fever in the developing world. In the sub-Saharan Africa RHD still is common and does not seem decreasing while HHD and IHD are rising competing with the already existing RHD as cause of heart disease in the area “ double burden of pre-transitional and post transitional diseases” (4, 7– 9).

Ethiopia as one of the African countries also shares the burden of cardiac disease. Some studies conducted in the country indicated that the two major causes of cardiac disease are RHD and HHD furthermore IHD is on the rise (10, 11,12). Determining the current pattern of cardiac disease in our setting is required which can also be considered as the beginning of efforts to put forward in characterization of the most prevailing cardiac diseases in our setting and ultimately improve its detection, treatment and possibly prevention (4). Therefore, this study was carried out to describe pattern of cardiac diseases among patients who started follow-up at Jimma University Specialized Hospital (JUSH) cardiac follow-up clinic.

## Methods and Materials

The study setting is Jimma University Specialized Hospital which is located in Jimma town 350Km Southwest of Addis Ababa. It is the only specialized hospital in Jimma zone serving the majority of peoples living in Jimma City and its surrounding. JUSH gives both in patient and out patient services. As one of the out patient services, the hospital has specialty clinics where patients with specific chronic disease are referred for follow-up. These clinics are staffed with internist, residents and nurses who are trained in specific chronic disease patient follow-up.

A cross-sectional study was employed to describe pattern of cardiac diseases among patients registered and started follow-up at the cardiac referral clinic of JUSH during the period from September 14, 2003 – September 14, 2008. The data collection was conducted from June 10, 2008 till October 9, 2008

The source population for the study was all adult cardiac patients registered at JUSH cardiac follow-up clinic during the period from September 14, 2003 – September 14, 2008. The study population was all the source population whose records were available. Sample size was not determined as all the source population was included in the study.

Data were collected using structured pretested format by a first year Internal Medicine Resident. The format included the card number, the date when the patient get registered to the cardiac follow-up clinic, card number, socio-demographic variables, and patient diagnosis including investigations regarding to the underling cardiac disease etiology. The study participants were selected by their name and card number from the cardiac clinic patient registration book, then individual patient's card was traced and collected to fill the data collection format.

The collected data were cleaned, edited and entered into SPSS for Windows version 12.0. Afterwards it was analyzed using SPSS for windows version 12.0 statistical software program. Descriptive analysis was made to describe the pattern of disease and associations were assessed using Chi-square test. Results were then presented using summary measures and frequency distributions. Associations with p-value <0.05 were considered to be significant.

The data quality control measures undertaken include: pre-testing of data collection tools, training of data collectors and supervisors and checking completeness and internal consistencies of data.

Ethical clearance was obtained from the department of internal medicine, Jimma University. Permission was obtained from the hospital officials before data collection.

## Results

Out of the 837 cases with cardiac compliant the records of 56 patients were incomplete for the etiologic diagnosis. As a result a total of 781 newly enrolled cardiac patients' record information was analyzed.

Among the total of 781 newly registered cardiac patients 409 (52.4%) were males and 372 (47.6%) were females. The mean age of patients at the time of enrollment was 43.5 (SD±17.2 years. Four hundred (51.9%) of patients were from rural, 254 (32.9%) from urban and 117(15.2%) from semi urban (Table 1).

**Table 1:** Socio-demographic characteristic of cardiac patients registered and started follow up at the cardiac clinic of JUSH, during the period from Sep 14, 2003 – Sep 14 2008.

Variables		Frequency	Percent
Sex			
	Male	409	52.4
	Female	372	47.6

Residency*			
	Urban	254	32.9
	Semi urban	117	15.2
	Rural	400	51.9

* Residential area was not documented for 10 patients.

Out of 781 cardiac patients 256 (32.8%) had RHD, 189(24.2%) HHD 158(20.2%) cardiomyopathy, 94(12.0%) IHD, 30(3.8%) Cor-pulmonale 27(13.5%) arrhythmia and 27(3.4%) had other sorts of heart diseases (Table 2).

**Table 2:** Cardiac disease distribution among patients registered and started follow up at the cardiac clinic of JUSH, during the period from Sep 14, 2003 – Sep 14 2008.

Type of heart disease		Frequency	Percent
	RHD	256	32.8
	HHD	189	24.2
	Dilated	158	20.2
Cardiomyopathy			
	IHD§	94	12.0
	Cor-pulmonale	30	3.8
	Arrhythmia	27	3.5

	OthersΨ	27	3.4

§ 52 patients were hypertensive and 13 were smokers.

Ψ Pericardial disease, congenital heart disease, degenerative valvular disease

The mean age in years for the respective cardiac disease at the time of registry was 31.4 (SD±13.2) for RHD; 51.1 (SD1±4.4) for HHD, 46.5 (SD±16.0) for cardiomyopathy, 57.2 (SD±12.2) for IHD, 47.0 (SD±16.5) for Cor-pulmonale and 33.7 (SD±16.2) years for Arrhythmia. There was statistically significant difference in mean age of patients with different types of cardiac disease (P<0.001). Statistically significant mean age difference between male and females patients was observed among HHD (p=0.002) and cardiomyopathy (p=0.004) patients only (Table 3).

**Table 3:** Mean age distribution by sex of cardiac patients on follow up at the cardiac clinic of JUSH, during the period from Sep 14, 2003 – Sep 14, 2008.

Etiology		Sex			P Value*
			
		Male	Female	Total	
RHD					
	Mean age	1.7	31.1	31.4	0.737
	SD	14.1	12.4	13.2	
HHD					
	Mean age	53.6	47.0	51.1	0.002
	SD	14.7	13.1	14.4	
Cardiomyopathy					
	Mean age	50.1	42.9	46.5	0.004
	SD	16.7	14.4	16.0	
IHD					
	Mean age	58.0	56.0	57.2	0.437
	SD	11.3	13.5	12.2	
Cor-pulmonale					
	Mean age	44.2	49.1	47.0	0.43
	SD	18.6	14.9	16.5	
Arrhythmia					
	Mean age	34.8	32.5	33.7	0.717
	SD	17.3	15.5	16.2	
Others					
	Mean age	54.8	40.1	45.6	0.041
	SD	14.8	18.2	18.2	
Total					
	Mean age	46.3	40.4	43.5	0.000
	SD	17.9	15.9	17.2	

P-valueΨ				0.000	

* p-value from independent sampling t-test

Ψ p-value from ANOVA

Concerning distribution of sex, males constitute 118(46.1%) among RHD, 118 (62.4%) among HHD, 79 (50.0%) among cardiomyopathy, 57 (60.6%) among IHD, 13(43.3%) among Corpulmonale and 14(51.8%) among arrhythmia patients (Table 4).

**Table 4:** Cardiac disease distribution by sex and residency among patients registered and started follow up at the cardiac clinic of JUSH, during the period from Sep 14, 2003 – Sep 14 2008.

Etiology	Sex			Residential area	

	Male	Female	Urban	Semi-urban	Rural
RHD	118 (46.1%)	138 (53.9%)	76 (30.0%)	39 (15.4%)	138 (54.5%)
HHD	118 (62.4%)	71 (37.7%)	63 (33.7%)	40 (21.4%)	84 (44.9%)
Cardiomyopathy	79 (50.0%)	79 (50.0%)	43 (27.9%)	18 (11.7%)	93 (60.4%)
IHD	57 (60.6%)	37 (39.4%)	39 (41.9%)	12 (12.9%)	42 (45.2%)
Cor-pulmonale	13 (43.3%)	17 (56.7%)	15 (50.0%)	3 (10.0%)	12 (40.0%)
Arrhythmia	14 (51.9%)	13 (48.1%)	11 (40.7%)	1 (3.7%)	15 (55.6%)
Others	10 (37.0%)	17 (63.0%)	7 (25.9%)	4 (14.8%)	16 (59.3%)
Total	409 (52.4%)	372 (47.6%)	254 (32.9%)	117 (15.2%)	400 (51.9%)
P-value*		0.005		0.025	

* p-value from Chi-square test

Among 256 RHD patients 193 (75.4%) patients had Echocardiographic report for the valve(s) involved during the time of enrollment and in the remaining RHD diagnosis were based on clinical and X-ray evidence. The distribution of valvular lesion based on echocardiography included isolated mitral stenosis (MS) in 99 (51.3%), combined MS and mitral regurgitation (MR) 49(25.4%), isolated MR and isolated aortic regurgitation (AR) each 12(6.2%), combined AR and MS 11(5.7%), and others 10(5.2%). Concerning sex distribution of patients with major types of valve abnormalities, 44 were males and 55 were females among patients with isolated MS and 22 were males and 27 were females among patients with combined MS and MR. However, the difference in sex distribution was not statistically significant (P= 0.388) (Table 5).

**Table 5:** Pattern of valve(s) involvement among RHD patients registered and started follow up at the cardiac clinic of JUSH, during the period from Sep 14, 2003 – Sep 14 2008.

Echocardiography report of valve(s) involved	Sex		P-value*
	Male	Female	
MS	44(44.4%)	55(55.6%)	
MS+MR	22(44.9%)	27(55.1%)	
MR	6(50.0%)	6(50.0%)	
AR	8(66.7%)	4(33.3%)	0.388
AR+MS	6(54.5%)	5(45.5%)	
MR+MS+AR	3(42.9%)	4(57.1%)	
Others	0	3(100%)	
Total	89(46.1%)	104(53.9%)	

* p-value from Chi-square test

## Discussion

This study was carried out to assess the pattern of cardiac disease in the context of ongoing global epidemiologic transition to which Ethiopia is no exception. The pattern of cardiac disease at JUSH where this study is conducted was assessed 5 years back on a sample of 58 patients. The study reported that 72.4% of cardiac patients were having RHD (13), which should have been an alarm for further studies which need to include screening of high risk individuals so that actual disease prevalence could have been better estimated and subsequent measure could have been done. However, no further studies have been done then after.

The finding of this study would not represent all cardiac cases visiting the hospital as cardiac patients with co-morbid diabetes mellitus were followed at the diabetic clinic and the hospital lacks diagnostic facilities such as cardiac markers. The study was undertaken with such limitations.

The leading four cardiac disease etiologies identified in this study were RHD, HHD, cardiomyopathy, IHD. The finding is similar as compared with the result of other studies done in Ethiopia and Malawi (10, 11, 14). In the study conducted on the same setting 5 years back, the pattern of cardiac disease in order of decreasing frequency was RHD, Cardiomyopathy, HHD and others (13). The difference could be partly due to difference in case definition and sample size.

Rheumatic heart disease was found to be the leading cause of cardiac disease which is similar with the finding of a study conducted 5 years back on the same setting but the proportion in this study (32.8%) is lower than the previous (72.4%) (13) which could be due to the actual increase in the other type of heart diseases and partly the difference in the methodology described above may also contribute. It was found that symptomatic RHD patients presented at mean age of 31 years affecting the productive group of the community. Studies showed that lower mean age at development of the diseases was 23 years in Gondar, 25.5 years in Tikur Anbesa Hospital and 24 years in Nigeria (10, 15, 16). In countries like Ethiopia where surgical intervention for cardiac disease is unavailable adverse outcomes including disability and death of such groups of patients is imminent and the loss to the countries is significant.

Among the RHD patients who had Echo report the Pattern of valve involvement was comparable with other studies (11, 17). Overall, MS which is a chronic complication was the identified in 86% of patients at the time of first presentation. The presence of MS is evidence for long asymptomatic period which could potentially be intervened by secondary prophylaxis if identified earlier (18).

Hypertensive heart disease was the second commonest (24%) type of heart disease. This is higher as compared to the finding of the previous study done at JUSH (12%)(13). In other studies done in developing countries hypertension has become the 1^st^ or the 2^nd^ leading cause of heart disease in the latter case with a progressive narrowing in the magnitude difference between RHD and HHD (10, 11, 12,19). Female patients with HHD presented at younger mean age than males. This could be partly due to the fact that males tend to be more active than females ; physical activity have a positive effect with regard to hypertension prevention and it control so at least it delays its progression as found in the South African study (20) which may be the case in our set up too. This issue however needs further study.

Dilated cardiomyopathy accounted for a fifth of cardiac patients and it was the 3^rd^ commonest type of heart disease. Similar findings were noted from similar studies done in developing countries including the previous study done on the same setting (10, 13, 14). Ischemic heart disease was the fourth leading type of cardiac disease. Majority (69%) of them had at least one of the conventional risk factors (hypertension or smoking) of CVD. The proportion of IHD and conventional risk factors is similar with findings of other studies (14, 21, 22). Therefore identification of cardiovascular disease risk factors and determination of its overall prevalence in our community is required so that both individualized and community based interventions could be implemented (4, 23).

In conclusion, this study has found that three-quarters of cardiac diseases were due to RHD, HHD and cardiomyopathy in decreasing order of frequency. Statistically significant difference in age at presentation was observed where RHD occurred in the younger productive age group. The proportion of patients with hypertension as cause or contributing factor for cardiac disease has increased. As this study is hospital based with limited representativeness population based studies are recommended
